# Family and community care for older persons with Parkinson’s disease in Kenya: a qualitative, ethnographic study

**DOI:** 10.1186/s12877-026-08051-z

**Published:** 2026-07-27

**Authors:** Natasha Fothergill-Misbah

**Affiliations:** Population Health Sciences Institute, Newcastle University, Newcastle upon Tyne, UK

**Keywords:** Reciprocity, Intergenerational, Africa, Ageing, Gerontology, Disability

## Abstract

**Background:**

Informal family care remains central to care for older adults in Kenya, yet evidence hints towards an ‘erosion’ of large, multi-generational family care networks and a conditionality on reciprocity across many African countries. The increasing incidence and prevalence of chronic conditions associated with later life have made care needs more complex and resource intensive. Parkinson’s is one such example of a progressive condition characterised by a host of motor and non-motor symptoms, which results in increasing disability over time and, therefore, care requirements and resources. The experience of older persons experiencing progressive disability, and the impact on family care, is under-reported in Kenya and across Africa.

**Methods:**

This paper reports an ethnographic study on the lived experiences of older persons with Parkinson’s (aged 60 and older) and their families living in urban and rural Kenya, drawing on interview material and observations involving 45 people with Parkinson’s and 23 family caregivers to explore the experiences of receiving and providing care.

**Results:**

The paper explores care provision and receipt in the context of ageing with Parkinson’s as a progressive movement disorder, demonstrating the resilience and adaptability of older adults and their caregivers. Structural, cultural and community processes situate the individual experiences of PwP and family caregivers, who navigate taboos and tensions in reciprocity and resources to make care work. These experiences have implications for who takes on the caring role, and the resulting adaptations, reconfigurations and emerging forms of care that families enact, including unconventional co-residence.

**Conclusions:**

Older people with Parkinson’s described contending with the progressive nature of Parkinson’s and the erosion of independence, while informal caregivers reported the physical, emotional and financial burden of caregiving. Care work was becoming more difficult, yet families in this study demonstrated adaptability, resilience and enduring obligation to enact and reciprocate care. This study emphasises the need to strengthen, support and sustain community-based approaches to care for older people in Kenya, and elsewhere in Africa. This need is particularly acute as the population rapidly grows and ages, and more older people age with, and into, disability.

## Introduction

Parkinson’s is the 2^nd^ most common neurodegenerative disorder globally, with evidence of rapidly growing prevalence [1, 2]. The progressive movement disorder is associated with motor and non-motor symptoms that result in increasing disability over time [3]. Motor symptoms involve tremor of the hands and feet, rigidity (muscle stiffness), slowness of movement, and loss of balance [3]. Non-motor symptoms vary hugely and often have the biggest impact on people with Parkinson’s (PwP) and on caregivers. These can include psychiatric changes (depression, anxiety, apathy), cognitive dysfunction (which can progress to dementia), sleep disturbances, autonomic nervous system issues (e.g., constipation, urinary incontinence), pain and other physical changes (e.g., difficulty swallowing or soft speech), among others. These varied and challenging symptoms, coupled with the advanced age of most PwP, results in individuals with increasing care requirements, particularly in later stages of disease. Therefore, the role of care networks and family becomes crucial when making sense of the experiences of Parkinson’s in later life. 

In Kenya, awareness about Parkinson’s is low. Poor awareness coupled with an absence of policy [4], limited neurological workforce capacity [5] and inaccessible medication [6, 7] contributes to delayed diagnoses [8], poorly managed symptoms, and perceptions associated with supernatural beliefs [9], perpetuating stigma and sometimes resulting in severe community discrimination [10, 11].

### Demographic transition and epidemiological shifts in Africa

Understanding the experience of ageing with Parkinson’s in Kenya requires consideration of the demographic changes occurring in Kenya, and elsewhere in the region. These ongoing transformations frame this work at a macro level, including rapid population growth and ageing [12], an increase in the prevalence of chronic disease [13], rural-urban migration [14, 15], shifts in the availability of informal care networks [16], an absence of health insurance and formal social support [17], and enduring poverty.

Almost 160 million people will be aged over 60 years in sub-Saharan Africa by 2050, growing to 630 million by 2100 [12]. As of 2024, 2.7 million people aged 60 and over live in Kenya [12], 84% of whom live rurally [18]. However, ageing continues to receive little attention and priority on government agendas [19], despite global initiatives such as the UN Decade of Healthy Ageing 2021–2030 [20]. Since the 2002 political declaration of the Madrid International Plan of Action on Ageing (MIPAA), implementation of policy to meet the challenge of demographic transition has been lacking on the continent, including in Kenya [21, 22]. McQuaid et al., for example, discuss how older (urban) populations in Africa are excluded from decision-making and agenda-setting because of the flawed assumption that older people are inactive and/or unproductive [23].

Populations in Africa are also facing an increasing incidence and prevalence of chronic, non-communicable diseases, including neurological disorders [24]. Data from the Global Burden of Disease study show that Parkinson’s is the 5th and 3rd leading cause of global disability adjusted life years (a measure of burden of disease) across all neurological disorders in the 60–79 years and 80 + years categories, respectively. The prevalence of Parkinson’s in these age groups is 431 per 100,000 population and 1,773 per 100,000 population, respectively.

There are reported to be almost 12 million people living with Parkinson’s globally (an increase of 274% since 1990) [25, 26], though true prevalence is difficult to determine, particularly in low- and middle-income countries, due to under-diagnosis and inconsistencies in reporting. For example, Fothergill-Misbah et al. have described how healthcare professionals in Kenya dismissed Parkinson’s as something that only affects ‘Western populations’ [8]. However, Su et al. have reported 90,000 people living with Parkinson’s in Eastern sub-Saharan Africa, with projections this will grow to 311,000 by 2050 due largely to population ageing and overall growth [27]. This projected growth in the number of people with Parkinson’s is the 2nd highest predicted change of all world regions, only behind Western sub-Saharan Africa. These are likely underestimations based on the limitations noted above. Community-based prevalence studies could support more accurate estimations of prevalence across Africa [28]. Considering the substantial growth of populations in Africa, the burden of Parkinson’s is going to be significant.

The impact of health conditions in later life can also be partly attributed to the accumulation of vulnerabilities across the life course – in access to healthcare, education, income generating activities, social protection and insurance – which play key roles in shaping the lives of individuals through to old age [29]. The WHO Africa region has the lowest healthy life expectancy at birth of all world regions [30] and older adults aged 60 and above on the continent experience the highest rates of years lived with disability [31].

### Care structures for older people in Africa

Informal family care described throughout this paper refers to ‘direct, personal and relational care work’ (e.g., feeding or bathing), ‘indirect care activities’ (e.g., cooking or cleaning), as well as medical/nursing care and financial care [32]. A systematic review by Hailu et al. on the role of family caregivers in supporting older adults in Africa describes the physical, emotional and health related support provided to older persons, particularly those with chronic diseases (the authors refer to diabetes, hypertension or dementia) [33]. These multiple roles are often taken on without support or training and take their toll on family caregivers who experience physical, financial and psychological strain. A systematic review on the experiences of caring for older persons with dementia in sub-Saharan Africa identified similar impacts on caregivers in addition to unmet needs, compounded also by the stigma and discrimination associated with having dementia [34]. However, caregivers were able to seek support from wider family units, community groups and, where available, dementia support groups.

The family remain central to care for older people in Kenya [35] – a norm reinforced by governments and policies in many African countries [36, 37]. Care is often described and seen as a collective responsibility with traditional care structures across Africa embedded in complex cultural and historical systems where older people are seen as both givers and receivers of care [14] – ‘reciprocity’ is the cornerstone of family support. In Kenya, ‘success’ in old age has been described as being amongst kin and *partly* dependent on assistance, whilst also being needed by family and community [38]. Whyte [39] echoes this view in Uganda, where she argues decline is an accepted and expected part of life that brings the opportunity of *interdependent* care. This “desired interdependence” suggests a more interconnected structure of family and community care that offers opportunities for fluid care. Kimuna, for example, has reported how older adults in Zimbabwe choose living arrangements based on their needs [40]. Mwendwa et al. note, however, that this is contingent on familial bonds and can be conditional on the family’s understanding of the health condition (e.g., dementia) [34].

Despite this reliance on family care in later life, African gerontological literature hints at the ‘erosion’ of large, close-knit families and the emergence of a conditionality on ‘reciprocity’ in intergenerational care [37, 41–43]. Economic tensions are making informal, family care more difficult [44]. Formal care (including care homes or residential care), however, remains practically non-existent across much of the continent, and highly stigmatised [45], while support for community-based informal care is also poor. As care structures shift [43] and rates of disability increase – requiring increasing direct care and resources – older people may not always have a say in where, or with whom, they live, or in the support they receive.

Changes in the availability of community support networks is therefore resulting in a ‘care deficit’ [16], where reciprocity is expected and respected yet is competing with wider structural forces. This deficit in the availability of family care has been shown to result in reconfigurations of care [46]. For example, evidence of changing forms of care, including ‘care from a distance’, emerges from Ghana [47], while in Tanzania, Obrist refers to ‘kin-based carescapes’ where family provide monetary contributions, advice, material help or medication from a distance [46]. These forms of care appear less conventional than the traditional multi-generational ‘direct’ care previously seen and expected but seem to be practical solutions to social change, while enabling older persons to maintain self-identity. Understandings and explanations of such experiences are needed to respond to the context-specific needs of communities and populations across Africa [48].

Therefore, the aim of this study is to explore the experiences of receiving and providing care from the perspective of older people living with Parkinson’s and their families in Kenya. Specific objectives are as follows:

1. To understand how older people with Parkinson’s experience informal family care within the home and community.
2. To understand the role of informal family caregivers and the impact of the caregiving role.

## Methods

### Design

The data presented in this paper come from an ethnographic study exploring the lived experience of Parkinson’s in Kenya carried out over 10 months in 2018/2019. Most fieldwork took place in Nairobi and Mombasa, two major cities in Kenya, and their surrounding rural areas, with additional study periods in rural central Kenya. Ethnography as a methodology can be used to acquire a holistic and in-depth insight into people’s lives, social interactions, behaviours and perceptions [49].

### Data collection

Fieldwork involved the author undertaking in-depth interviews with PwP aged 60-years and above (*n* = 45) and family members who cared for older PwP (*n* = 23), in addition to ethnographic observations in participants’ homes.

#### In-depth interviews

Semi-structured topic guides took a biographical approach, covering the following areas: journey to diagnosis; disease management; symptoms, informal care and social relationships; and the end of life (where applicable). Initial interviews (*n* = 45) with PwP were not audio recorded to avoid the risk of losing rapport. Instead, detailed hand-written notes were taken. In many cases, a family member was present with PwP during their interviews, depending on individual preference, level of PwPs’ cognitive or speech impairment, and the relationship of the caregiver. Sometimes, caregivers’ presence allowed for a richer description of experiences, though sometimes also meant PwP could not express their experience of care as freely as they may have liked, which is a potential limitation of the data.

Nine follow-up interviews were conducted with PwP one-on-one – these interviews were audio recorded (once rapport had been established) and took place when there was scope for further exploration after preliminary analysis, or when a participant’s circumstances had changed over the fieldwork period, e.g., living situation or change in symptoms.

All interviews with family members were audio recorded, and no PwP were present during interviews with family members, allowing them to discuss their experiences freely. Four of these interviews involved two caregivers (e.g., two sisters who cared for their mother together, or a spouse and daughter). Therefore, 19 interviews took place with 23 caregivers.

A total of 73 interviews took place – 45 PwP at baseline, 19 with caregivers, and 9 follow-ups with PwP. Almost all interviews were conducted in English; 10 interviews with PwP took place in Kiswahili and required an interpreter. Interviews took place in different locations, depending on the preference of the participant, including in people’s homes, at clinics or in cafes. Participants all provided written, informed consent to take part in the interviews. 

PwP were identified and invited to the study via two avenues. Firstly, through neurology clinics in Nairobi (public and private) and Mombasa (private). At each clinic, the author identified PwP through clinic files, with permission and support from staff, and invited PwP to participate. PwP were reminded that their participation was voluntary and would not affect their current care. Having said that, power imbalances may have influenced some to participate in the hope that they might receive additional care or knowledge through the research. Secondly, participants were recruited through an existing support group for Parkinson’s in Nairobi. The author attended monthly meetings and described the research to attendees, who were invited to approach the author if they were interested in participating. Family members were invited to participate in the study through both avenues – directly through the support group, and with the consent of PwP recruited from clinics. By virtue of these methods of recruitment, the sample is necessarily skewed to PwP with sufficient resources to access care, illustrating a potentially more optimistic picture of care for older PwP in Kenya – this is addressed further in the limitations.

#### Participant observations

The author also undertook observations in the homes of PwP and caregivers (totalling 66 h) during home visits for interviews. Field notes were handwritten and provided detailed and nuanced descriptions of the spaces, living situations, details of surrounding areas, relationships between PwP and family members, how PwP manoeuvred their homes, emotions, actions, and events that took place [49]. Observations in PwPs’ homes facilitated an understanding of PwPs’ changing state through the day as their medication took effect or wore off and the impact on their ability to carry out activities and communicate.

Observations, personal reflections and interview data underwent triangulation to test the accuracy of data and quality of information [50]. For example, Caroline (pseudonym) described her husband’s memory loss as a “big issue” during our interview. Throughout my time visiting their home, Maina, her husband with Parkinson’s, appeared absent and distracted, and later could not recall his birth date when I asked him how old he was.

### Data analysis

Thematic analysis was used to facilitate an in-depth understanding of the data, helping to identify underlying patterns and meaning, while aligning with the interpretative nature of ethnography. Analysis was iterative, broadly following Braun and Clarke [51] six phases, with constant reviewing of transcripts, as well as personal reflections and observation notes while in the field to support the data. Post-fieldwork, coding of hard copy interview transcripts was carried out by the author, with 10% of interview material coded by a second experienced qualitative researcher (not involved in the data collection process) to check for reliability and subjectivity. Conceptualised empirical and theoretical codes were reviewed and collated into themes; this process utilised diagrams, mind maps, and reviewing manually coded data across codes. Themes were reviewed collectively and considered in the context of the data, particularly whether these holistically captured the lived experience of PwP and caregivers in Kenya. Finalised themes were named and described before being presented alongside supportive quotes. For example, codes around the multiple caregiving tasks formed the theme ‘It becomes your life’, reflecting the full-time role described by caregivers. Quotes in the findings support the themes and broadly reflect the experience of participants. All names used in the findings are pseudonyms. Reflexivity throughout the process was crucial, facilitating reflections on ideas, experiences and biases [52].

### Ethical considerations

Ethical approval to conduct this study was obtained from the Faculty of Medical Sciences Research Ethics Committee, Newcastle University, UK (Application No. 1293/14933/2017), from Kenya Medical Research Institute (KEMRI) Scientific Ethics Review Unit (SERU) (Reference: NON-KEMRI 609) and Kenyatta National Hospital – University of Nairobi Ethics and Research Committee (KNH-UoN ERC) (Reference: P451/06/2018). All participants in this study gave full informed consent to participate (see Declarations).

### Description of sample

Table 1 outlies the demographic characteristics of the 45 older PwP included in this study (aged 60 and above). PwPs’ ages ranged from 60 to 81 years old with an average age of 69.5 years. Thirty PwP lived in Nairobi and Mombasa while 15 PwP lived rurally (either in the surrounding rural areas of the cities or in rural central Kenya). The heavy skewing towards urban proximity results from the accessibility of neurology clinics. On average, PwP lived with 3 other people – this ranged from living alone, to one PwP living with 15 family members. Most PwP were not employed at the time of study, while just over half did not have a pension scheme. Though not systematically recorded, many PwP reported living with multiple long-term conditions, most commonly diabetes and hypertension, while several PwP experienced bouts of pneumonia as a result of their Parkinson’s.

**Table 1:** Description of PwP characteristics (*n* = 45)

Sex:	Male (*n* = 26)
	Female (*n* = 19)
Age range:	60–64 (*n* = 11)
	65–69 (*n* = 13)
	70–74 (*n* = 9)
	75–79 (*n* = 10)
	80+ (*n* = 2)
Residence:	Urban (*n* = 30)
	Rural (*n* = 15)
Number in household:	Living alone (*n* = 3)
	Cohabiting with 1 person (*n* = 14)
	Cohabiting with 2–3 persons (*n* = 13)
	Cohabiting with 4 persons (*n* = 7)
	Cohabiting with 5 + persons (*n* = 8)
Caregiver status:	Main caregiver (*n* = 29)
	No main caregiver (*n* = 16)
Caregiver sex:	Female (*n* = 20) Male (*n* = 9)
Caregiver relation:	Spouse (*n* = 14)
	Daughter (*n* = 4)
	Employed caregiver (*n* = 4)
	Son (*n* = 3)
	Grandchild (*n* = 3)
	Niece (*n* = 1)
Employment status:	Not employed (*n* = 31)
	Self-employed (*n* = 9)
	Formal sector (*n* = 4)
	Public sector (*n* = 1)
Pension status:	No pension (*n* = 27)
	Public service pension scheme (*n* = 10)
	Occupational scheme (*n* = 3)
	Private scheme (*n* = 3)
	National social security fund (*n* = 2)

Interviews with the 23 family members included twelve daughters, six sons, four wives, and one nephew, who all cared for PwP aged 60 and above – majority (*n* = 19) also lived in cities. PwP and family members were not necessarily interviewed as dyads. Eleven family members lived with their relative with Parkinson’s and seven either lived nearby and visited PwP often or provided financial care from a distance. Five family members of PwP who had died prior to the study were also interviewed.

## Results

Analysis of the interview data generated three key themes related to the topic of ‘care’. The first theme *‘Care receipt and care provision’* explores the impact of Parkinson’s on individuals and their families, highlighting the increasing requirements of care and the evolving role of ‘caregiver’. The particular context of Parkinson’s – and perhaps other progressive neurological conditions – is crucial in framing the lives of older PwP and the implications for how they age. The second theme *‘Structural*,* cultural and community processes’* situates the individual experiences of PwP and caregivers within broader structural processes (urban migration and absence of formal care), cultural processes (tensions in reciprocity and avoidance rules), and community processes (community care networks), to examine the influence on care provision and receipt. The third theme ‘*Reconfigurations of care*’ discusses the implications for where PwP were able to live and, crucially, to receive the care they needed. Pseudonyms are used for all participants throughout the findings.

### Theme 1: Care receipt and care provision

#### *“It’s a disease which destroys”*: experience of living with Parkinson’s

The relentless progression of Parkinson’s symptoms affected the ability of older PwP to maintain independence and autonomy. PwP became both physically unable to carry out once ‘easy’ tasks, but also increasingly reluctant to engage in activities or social occasions due to low mood and apathy. Challenges with speech and communication (affecting social interactions), chewing and swallowing (resulting in assistance with mealtimes), falls at home and in public (resulting in fear of falling and of judgment by onlookers) and memory loss (requiring external involvement in tasks) were common.

During a visit to Caroline and Maina’s home in Nairobi, the progression of Parkinson’s became acutely apparent when Maina, a person with Parkinson’s, could not remember his date of birth, rolling off a series of incoherent numbers, which frustrated his wife. At aged 68, Maina’s cognitive difficulties were his most noticeable symptom. Caroline described how Maina, for example, was mixing up his cutlery, using a spoon instead of a knife to cut meat. Maina also fell later in my visit, missing the sofa as he lowered himself down.

Consequently, several PwP described feeling they were becoming a “burden” on their families. Family members, in turn, reported trying to enable PwP to remain independent. Esther, who provided care from a distance to her 78-year-old mother with Parkinson’s, Leah, explained how buying her a wheelchair meant she could see her whole house for the first time in years. Some PwP welcomed this support, while others viewed the extra help they needed as a “weakness” or symbol of how “helpless” they were, denying assistance. For example, Leah repeatedly tried to fire her employed caregiver, despite being unable to walk, or make herself a cup of tea.

On visiting Paul, a 70-year-old retired lecturer with Parkinson’s living on the outskirts of Nairobi, the importance of maintaining some independence became acutely apparent. Paul was annoyed about his condition, exclaiming, “*This disease cannot allow me to do many things”*. He added it was disheartening that he struggled to turn over in bed or stand up from the sofa: *“I want to be independent; I don’t want to keep calling people”.* Throughout my stay, Paul repeatedly attempted to stand up from the sofa and struggled, insisting that he could get up and walk independently. At times he looked like he might fall but made it back to the sofa. Later, he dropped down to the ground and performed two push-ups on the carpet. He proudly confirmed that he did two every day.

The progression of Parkinson’s symptoms resulted in an increasing need for assistance or help (initially) and, eventually, the need for ‘care’. Richard, a 68-year-old PwP lived with his wife, daughter and paid carer in a second-floor apartment in Nairobi, accessible only by stairs. He told me that he had a fear of falling and his daughter insisted he wear a whistle after he had fallen twice. Richard described getting stuck between the toilet and bath in his home. Unable to get himself un-stuck, he rang his whistle for help.

Associated with the extra need for care was a shift in the roles of PwP and family members, which often came with some pushback from PwP. For example, PwP described struggling to accept that their adult children were becoming their caregivers. Outside the family, participants also described difficulties maintaining social roles within their communities.

#### *“It becomes your life”*: experience of caring for someone with Parkinson’s

As the physical assistance required by PwP became more complex, caregivers reported their increasing roles and responsibilities over the years. For example, Constance cared for her husband with Parkinson’s in their rural coastal home for 14 years:

*“The whole night*,* in fact*,* I’m a nurse*,* night duty. When you go back to your bed and try and settle*,* he calls you back. I am very tired because of the day’s work; I want to rest. After every 5 minutes he wants to urinate*,* then urinate there on the bed then I will wash the bed sheets. So*,* we quarrel every night. Then I wash and remember*,* ehh*,* washing the bed sheets is more difficult than lifting him up. So*,* we have a story every night”* (Constance, wife of 70-year-old PwP)

The degree to which caregivers experienced physical, emotional and financial strain was largely dependent on social and financial resources. Social support varied; some families took on the caring role as a ‘team’, usually those with larger kinship networks and more opportunities for care, while others with no support networks described struggling with their role. Finding a balance between the caring role and their own life was difficult, with family members – often spouses or adult children – describing having *“given up”* or *“sacrificed”* their own lives. Pauline was in her 50s and lived with her mother, Leah, in rural central Kenya:

*“I used to have a shop and then my mum got sick. Now we started going to the hospital for clinics. At that time*,* I could still run the shop*,* but nowadays I am forced to close because most of the times*,* I’m in hospitals*,* I’m going for clinics. I have to make sure she gets the right dose at the right time. So*,* I had to close the business. So basically*,* that’s my life”* (Pauline, daughter of 78-year-old PwP)

The emotional strain of the caregiver role manifested through witnessing PwPs’ deterioration but mostly through doing care work, resulting in ‘burn out’. For example, Jenny and her husband lived in a Nairobi apartment with Jenny’s mother who was bed-bound because of her advanced Parkinson’s:

*“So*,* literally*,* we don’t have our lives. At some point your life has to stop*,* so that you can take care of her. At times*,* I feel I don’t want to come back home. I feel better when I go to take tea; I want to take more time there”* (Jenny, daughter of 77-year-old PwP)

Other family members described trying to negotiate their roles and not give up their own lives. Caregivers who lived with PwP (like Jenny) described their reliance on them for everything, while those who did not described experiencing worry and guilt when they were apart. There were also ‘expectations’ of what care should look like from outside the family, which came with judgments on the quality of care PwP received. Judith, a 69-year-old lady with Parkinson’s lived with her husband in the rural outskirts of Nairobi. Even though her son drove 45 minutes every day to see her and attended monthly Parkinson’s support group meetings, her extended family felt she had been ‘neglected’.

*“They wonder*,* ‘you’ve been to the doctor*,* and you’re not healed’? One of my sisters had to come here for two weeks just to understand what I’m going through. I learnt she was sent by my other sisters. That time she came*,* she found I had started stooping*,* she told me I should see another doctor”* (Judith, 69-year-old PwP)

A poor understanding of what Parkinson’s was and why it could not be ‘cured’ was common among extended families who blamed the family for not ‘doing enough’.

Financially, families reported using up savings, selling assets, borrowing money and holding fundraisers to source funds. Adult children in full-time paid work who could not physically care would instead provide financially. Esther, for example, had a good job in Nairobi, which meant she was expected to pay for all her mother’s care needs in rural Kenya, instead of physically caring for her. Esther identified herself as a caregiver and although she did visit her mother with Parkinson’s often, her main ‘role’ was to send money, medicines or ‘adult diapers’, while others did the ‘hands-on’ caring.

Although many PwP identified a ‘main’ caregiver, care networks were often complex and multi-layered, involving input from several family members and sometimes paid help, with different actors contributing to different needs, which varied temporally.

### Theme 2: Structural, cultural and community processes

#### Structural processes: migration and formal care

Extended, multigenerational families situated within large close-knit communities providing social capital and care opportunities for older people were perceived to be shifting, and ‘home’ was not what it used to be. Societal change appeared to be influencing the community networks and multigenerational family structures that once existed. These changes were articulated by one caregiver:

*“Nowadays*,* it’s not like before where you go to the village and there’s tons of people. There’s a lot of urban migration. A lot of people have their roots there*,* but they don’t live there anymore. There’s almost no one there. A lot of kids never used to go to school*,* now everyone goes to school. Younger people*,* what are you sitting around in the village doing? Probably makes more sense to go and get a job. If you stay in the village*,* how are you going to support your parents. A lot of the children left to go find work in the city and send money back. Sometimes there’s a relative who stays there in the village and the children just send money for food and sustenance. I think that’s the new dynamic*,* it’s happening all over Kenya”* (Danny, son of 83-year-old PwP)

The changing dynamics of rural life and family structures were thought to be creating difficulties for care, although care expectations were still very ingrained in families.

Participants spoke of care changing form. However, the option of formal long-term care was dismissed by most. Care homes are still scarce in Kenya, and often unaffordable, but crucially, there is a strong taboo and stigma associated with them. The idea of care homes was seen as shameful – one caregiver explained that older people would *“rather die than be sent to one of those places”*. Another suggested the following:

*“I think it’s as it should be that family care for older parents. It’s not even something that you’d suggest. It’s like ‘OK*,* you’re throwing me away*,* you’re dumping me’. We do have old people’s homes*,* but they’re not very popular. There’s a sort of stigma associated with them. ‘So*,* your kids dumped you here?’”* (Jasper, son of 78-year-old PwP)

Therefore, care continued to be carried out by families – at least for the participants in this study – although it was clear that this was becoming more difficult.

#### Cultural processes: reciprocity and avoidance rules

Participants explored their roles as caregivers and recipients of care, as well as the changing expectations of care for future generations. Reciprocal care was ingrained in families who had observed the care their grandparents had received from their parents and considered the care they received as children. Daughters and firstborn sons described feeling *“obliged”* to care for their parents with Parkinson’s or took on the role out of *“respect”*, with the expectation that reciprocity should be passed down through generations.

Some adult children who lived and worked in cities believed their parents with Parkinson’s could no longer expect the same physical, hands-on care they might have given their own parents. Others, particularly younger caregivers, suggested that the ability to care for their parents was changing because of changing material and financial circumstances.

*“It’s not reasonable to expect your kids to support you in old age. A lot of us feel that obligation*,* but life is expensive now. People are living longer*,* and you have kids who are struggling to get by in the city who also have their own future to think about. So*,* then they have that added burden of now having to take care of parents. But I think it was just ingrained in us. So*,* even if they don’t say*,* you just feel like you need to do it. There’s just a cultural thing*,* it’s just like kind of an expectation”* (Danny, son of 83-year-old PwP)

Although the *“obligation”* or *“expectation”* to care for parents was still present, in reality, care was becoming more difficult. Structural and societal changes were described as contributing to unstable care resources and eroding opportunities for intergenerational care.

‘Traditional’ and ‘cultural’ expectations of men and women within the family, and how gender influenced care also required negotiation. Most caregivers were, unsurprisingly, female, although male adult children described taking on active roles in their parents’ care. Most of the older male PwP were cared for by their wives, and female PwP were mostly cared for by adult daughters. However, daughters in cities described having responsibilities as wage earners and were increasingly becoming less able to physically care for older parents. Instead, they would provide financial care or employ caregivers (as Esther described), creating a shift in care structures.

PwP and their families described negotiating traditional ‘taboos’ which acted as barriers to care. ‘Avoidance rules’ [46], where only certain genders could perform certain roles, were explored, and sometimes dismissed. Jenny, caregiver of her 77-year-old mother with Parkinson’s, explained that, for example, it was culturally frowned upon for her mother to live with Jenny’s husband. However, she admitted, *“When such a thing [Parkinson’s] happens*,* it’s not important anymore. You cannot continue following those cultures when someone is suffering”* (Jenny, daughter of 77-year-old PwP). Other caregivers were clear that daughters could *not* provide intimate tasks for their father with Parkinson’s.

Families described negotiating these *“old school”* traditions to make care work, despite potentially unorthodox living arrangements (such as Jenny’s mother living in her home), while others stressed the importance of adhering to traditions (such as daughters not bathing their fathers). Some adult sons also appeared to be taking on physical care roles and negotiating gendered avoidance rules – often when there was no other choice. Jasper lived near his mother in the outskirts of Nairobi and often took on direct care roles in the absence of paid help:

*“Some things in my culture are taboo. For instance*,* taking mum to the toilet. It would be easier*,* I guess*,* for a girl. But these are things that need to be done. As mum says*,* ‘God knows us*,* so don’t worry’. We’ve sort of gotten into a routine. I put her on the toilet*,* she’s able to undress herself and I get out and*,* when she’s done*,* she calls me and I take her out”* (Jasper, son of 78-year-old PwP)

Gendered roles were being negotiated, and care was becoming more fluid in the face of increasing requirements, resulting in pragmatic decision making.

#### Community processes: community care networks

Participants spoke of the historical role of the community in care and support for ‘the sick’ or older people, particularly in villages. For example, Esther (caregiver) reflected on the community care networks available to her mother, Leah, in rural central Kenya:

*“You know*,* the best thing in rural Kenya*,* you are here in your house*,* but my brother and his family are somewhere there*,* my sister is somewhere there*,* my grandmother is there*,* even the small kids. It helps*,* that community thing helps”* (Esther, daughter of 78-year-old PwP)

The community played a crucial role in maintaining social networks for PwP, staving off loneliness, providing support and often for *Harambee*, a Kenyan tradition of fundraising which literally translates as ‘all pull together’. Participants also described the respect for older people in general where communities would help if the family were struggling, as per traditional customs.

However, several family members also described how PwPs’ community networks had begun to dissolve as they could no longer uphold expected social roles, like attending church or weddings, or visiting friends at their homes. Joseph, a pastor living with Parkinson’s in Nairobi, was still undertaking his ministerial duties, though added the following during our interview:

*“I feel sorry for myself. I don’t enjoy being around people. I feel like I want to excuse myself in social situations and avoid questions”* (Joseph, 65-year-old PwP)

Consequently, many PwP described having fewer visits from friends and family, increasingly leading them to feel out of place in their communities. PwPs’ masked faces (stiffness of facial muscles that reduces a person’s ability to express emotion) also led visitors to believe they were not happy to see them and, for some, beliefs that PwP were cursed or bewitched resulted in fewer social visits [9].

PwP living in cities reported trying to maintain ties with their rural communities and return ‘home’ as often as possible. The strength and support of the community in villages was one of the reasons PwP wanted to move back, although others acknowledged the changes happening to ‘rural life’. Gloria, a 76-year-old PwP, was living with her daughter and grandchildren in a ground floor Nairobi apartment. During an afternoon spent with her, she described how all the people of her age in her village had died and ‘nobody’ was left. The perceived shifts in ‘community’ in rural villages resulting from wider structural issues described earlier meant that the ability to return to rural life in old age was not a given.

Further challenges with the local environment included the scarcity of health services that PwP needed. Neurologists were in cities and, with the need for frequent follow-up consultations, living in the village was associated with significant travel costs and strain, particularly as individual mobility became more challenging.

### Theme 3: Reconfigurations of care

Initially, PwP and their families learnt to manage and get used to Parkinson’s, through making changes to everyday life and adapting to their new normal. This process required strong support networks, substantial resources and shifting roles – for some without families, adapting to Parkinson’s was more difficult. Crucially, this enabled PwP to live at home, with strategies including adapting the home to make their environments more liveable and manageable, for example, acquiring a hospital bed, adding railings to walls, moving bedrooms altogether, or creating pathways through the home. These changes were contingent on families’ financial situations. However, adaptations appeared to be short term solutions and required renegotiation as Parkinson’s progressed.

Participants described eventually having to make difficult decisions about where, and with whom, PwP lived to allow them to live as good a life as possible, given their circumstances. Wider structural shifts influenced the potential care for older PwP, particularly those living rurally. Sometimes this meant adult children moving to live with their parent in the village, if they were able, or PwP moving to the city with adult children to be closer to support and medical care. Relocations, however, were dependent on whether adult children could support their parent and/or were willing to relocate and/or negotiate avoidance rules. Such decisions were also often made by adult children rather than PwP themselves. Louise (caregiver) lived in Nairobi and described the decision process of relocating her mother with Parkinson’s.

*“Before*,* mum was living at home alone [in rural Kenya]*,* with one or two workers. But now we live with her*,* and it’s been much easier for everyone*,* including herself*,* because we see her now as often as we want. If she still lived there*,* she’d be gone by now. I don’t know who would have sacrificed to go live there with her. We are able to support her now from very close*,* so we are not spending a lot of hours travelling and now I think Nairobi has many good things*,* especially the support group meetings I think are helpful.”* (Louise, daughter of 71-year-old PwP)

Louise described the convenience of her mother moving to live with her in the city, as well as the proximity to helpful resources, such as clinics and support group meetings.

Other PwP who had moved to the city when they were younger described anticipating moving back to their rural villages after retirement, although this option became less ‘practical’ after the onset of Parkinson’s. The wider environment and neighbourhood could also make this more challenging as disability progressed due to the lack of pavements, bad roads, and lack of infrastructure for pedestrians and wheelchairs, resulting in many PwP rarely leaving their homes.

In some cases, PwP had no agency in the decisions made about their care or where they would live. Gloria had lived in rural Kenya and moved to Nairobi to live with her daughter nine years ago in search of a diagnosis and treatment. Gloria’s daughter wanted her to stay close to the services she believed she needed but Gloria wanted to return ‘home’:

*“I came to stay because of this disease*,* so now I feel let me go home and see what is there. Going back to the village is what I’m now thinking of. I can get all my medication and stay there.”* (Gloria, 76-year-old PwP)

The expectation of adult children to care for their parents with Parkinson’s remained evident among families of different socioeconomic status – those who had to physically care for their parents themselves and/or struggled financially, and those who could afford to pay for care. Several families described relying more on formal employed care in the home. The obligation to care was still there but for some wealthier, urban families care was changing form. For PwP who were cared for by spouses, relocations were less common, as they were (usually) able to be looked after in their own homes.

## Discussion

This paper explores the experience of receiving and providing care among older people living with Parkinson’s and their family caregivers in Kenya. Older PwP in Kenya are contending with a progressive condition that results in increasing disability over time, eroding independence and autonomy. Therefore, the role of the caregiver becomes central to PwPs’ experience in later life. What we see from this paper is how conditions like Parkinson’s push families to the limits of care, but also the resilience and enduring obligations of families to enact care and to enable older family members to maintain self-identity. Older PwP and their families described contending with increasing roles and responsibilities as Parkinson’s progressed, navigating expectations around reciprocity, intergenerational roles and avoidance rules, and the role of community networks in care. Practically, these experiences resulted in reconfigurations in care, often requiring adaptations and relocations.

As well as illustrating the empirical situation of care for PwP in Kenya, the data presented speak to wider theoretical discussions around care for older people in Africa, which are elaborated below.

### Identity and the limits of care

The progression of disability resulted in PwP struggling to maintain independence and a sense of ‘self’ [53], while also affecting their relationships and roles within the family, community and society. The findings illustrate how ‘easy’ activities of daily living can become ‘critical health moments’ in the context of later life with Parkinson’s – a term Obrist uses in Tanzania to describe how disability can impact the internal and external agency of older people [46]. However, we also see the crucial roles of strong connections and social resources in enabling older PwP to manage everyday life and to maintain a sense of identity, similar to the experience reported by Whyte among persons living with HIV in Uganda [54] who drew on kinship networks and friends to manage their chronic condition.

Despite family being the main source of care and support for older PwP in this study, many caregivers described the overwhelming and financially devastating care requirements of Parkinson’s, which could result in significant emotional and financial suffering, isolation and stress. The study demonstrates how care for PwP goes above and beyond the expected care requirements of older people (without a chronic, degenerative disease), requiring more input from the family, more pooling of resources and fluid care arrangements. Walga has identified similar experiences of shared caring responsibilities in Ethiopia among caregivers of PwP [55]. This finding raises issues when thinking about the epidemiological transition occurring in Kenya and across Africa, the increase in chronic disease associated with later life and the resulting care that older people may require for longer, particularly in the absence of formal social support provision. The physical, emotional and financial burden of caregiving related to Parkinson’s is not unique to families in Kenya [56–59] nor to older people in Africa more generally [33, 34]. However, this paper is the first to offer in-depth insights on care for older PwP in Africa, offering evidence to inform care practice and policy in Kenya (see section below).

### Tensions in reciprocity

The perspectives of caregivers in this study reinforced existing evidence that informal family care in Kenya is taking place in a context of social change where structural forces are influencing the availability of community networks, resulting in a ‘care deficit’ [16]. Through the narratives of caregivers, we see that reciprocity [47] continues to be expected, respected and upheld, yet is evidently competing with growing material and financial constraints on families, with potential influences on the availability and quality of informal family care. What became clear from participants in Kenya is that the increasing care requirements resulting from Parkinson’s strained the deep-rooted obligation of reciprocity and resulted in care potentially becoming more fluid as a result. Families therefore had to negotiate gendered and generational expectations of care in order to make care work for them, adapting to their unique circumstances. Aboderin [41] has noted similar constraints in care for older people in urban Ghana, with questions about whether families will be able to continue to uphold these expectations in the face of demographic and economic change. In Kenya, we see that a ‘reluctance’ to care for older PwP was not the dominant narrative, as has been hypothesised in the African gerontology literature [14, 60]. Care was just becoming more difficult, particularly in the context of Parkinson’s. Having said that, for those who never receive a diagnosis of Parkinson’s, these constraints may have indeed been completely disastrous (see limitations). When considering the perceived obligation of reciprocity, the impact on caregivers cannot be understated. Caring is both labour and financially intensive [33, 34], as described by caregivers in Kenya, although the extent of the burden has been relatively undocumented in the region [16, 61].

### Adaptability and emerging forms of care

The findings of this paper demonstrate the resilience and adaptability of older adults with Parkinson’s and their caregivers in shaping care practices. Families were navigating care while contending with wider structural processes, an absence of formal social support and unsupportive rural environments. These declines in support are not unique to Kenya [42] and have been reported in Uganda [62], Ghana [47, 63], Botswana [64] and South Africa [61]. Consequently, the paper shows how care for older people in Kenya is changing form and often resulting in reconfigurations in care and emerging forms of ‘new’ care.

Caregivers in Kenya reported initially adapting the home (if resources were available), often coupled with what van der Geest [47] has referred to in Ghana as “care from a distance”, or “kin-based carescapes” [46], where family ‘send’ monetary contributions, advice, material help or medication. Renegotiation often followed for participants in this study, with decisions about whether care would take place in PwPs’ own home, or whether relocation was necessary. The paper emphasised how decisions about reconfiguration (where, and with whom, PwP lived) often took place without consultation with PwP, mirroring observations by Obrist in Tanzania among older women who had to ‘accept’ the new arrangements made against their wishes [46]. Having said that, Hillcoat-Nalletamby and Ogg [65] suggest that, in the UK at least, dislikes about immediate home environment do cause older people to want to move – something that would merit further in-depth investigation among older persons in Kenya. In contrast, Cadmus et al. note how in Nigeria, an expectation of reciprocity contributed to a preference for older people to age in their own home [66].

Certain reconfigurations also involved families making pragmatic decisions, including negotiating gender taboos and ‘avoidance rules’ about who could perform certain roles. Through the data, we see how these renegotiations resulted in the formation of urban intergenerational households and ‘unorthodox’ living arrangements, including older women living with sons-in-law, or sons performing intimate tasks for their mothers. In contrast, strict adherence to gender and generation restrictions has previously been reported in coastal Tanzania [46] and Nigeria [66]. Perhaps the growing care needs of older people ageing with chronic and debilitating conditions, coupled with care deficits, will more often result in such emerging forms of care. Whether, and how, families increasingly negotiate taboos and rules to make care work (as seen in Kenya), as well as the implications for older people, require further exploration.

Participants also emphasised their reluctance to engage with formal, or institutional, care options, which remain limited and shrouded in taboos in Kenya and across Africa [67, 68]. Older people will undoubtedly be negotiating later life in the context of their changing bodies but also wider economic and social change and may increasingly need to rely on the state, market or self for care, rather than family [69]. Community-based care, however, offers a more practical, culturally sensitive mechanism to support families – emotionally, physically and financially – to continue to care for older relatives, whether rural or urban.

### Limitations

The experiences described in this paper were necessarily limited to those with a diagnosis of Parkinson’s. As such, one of the main limitations of this paper is that it represents a privileged population (in most cases) who had the social and financial resources to obtain a diagnosis – often a long, convoluted and expensive process in Kenya [8]. For those who never receive a diagnosis and who live rurally without connections to cities, the experience of Parkinson’s, PwPs’ opportunities for care, and the experience of caregiving are likely very different. Nonetheless, this paper offers important insights into the care experiences of a population under-represented in the literature on care and on Parkinson’s. Another limitation is the presence of family members during interviews with PwP – though this did not influence recollections of more clinical processes of diagnosis and treatment, it certainly could have prevented PwP from being open and honest about the quality of care they received, for example. However, many PwP were interviewed individually offering important (and potentially more honest) insights into experiences of care.

### Recommendations for future research

Future research could address the limitations noted above, particularly through exploring the care experiences of those PwP who never receive a diagnosis of Parkinson’s and who may experience additional precarity in the availability and quality of care. Further, longitudinal research could explore the shifts in care over time, particularly given the progressive nature of Parkinson’s, through to the end-of-life where care needs become even more complex. Further exploration of the consequences of social change on caregivers is required, particularly as government policies continue to advocate for filial care of older persons yet do not offer mechanisms for support. It would be important to look at the impact of neurodegenerative disease more broadly on older people and care opportunities in Kenya, incorporating the experiences of Parkinson’s and dementia, for example, to generate evidence for practice and policy.

### Policy and practice implications

Parkinson’s offers an important perspective of later life, associated with significant and progressive disability and care needs. Such experiences are becoming more common as life expectancy increases in Kenya, and across Africa, accompanied by chronic and potentially debilitating conditions – dementia [70], stroke [71], cancer [72] and frailty [73], to name a few. The impact of long-term chronic disease on older people and their families must be considered when developing future policies that incorporate the needs of older people and any national caregiver’s policies.

Despite the publication of a National Policy for Older Persons and Ageing in 2014 and review in 2018 [74], there are still significant gaps in the provision of care for older persons in Kenya. If families in Kenya are expected to continue to perform care in the community, they must be enabled to do so. Findings illustrate the need for financial support, potentially in the form of caregiver allowance; home-based care support to offer practical solutions in the home and respite to family caregivers; and community care services that enable older adults to continue to engage with their community, to maintain a sense of self and to mitigate loneliness – whether older people remain in place or relocate.

The ‘National Care Policy’ of Kenya [22], published in 2025, outlines key policy actions related to care for older persons and for persons with disabilities, encompassing many of the study’s policy implications outlined here. Though this is a positive step, it remains to be seen whether the implementation of this policy will make an impact on the lives of older people with Parkinson’s and their families.

## Conclusion

This research has contributed to the very limited understanding about the experience of care for older people living with Parkinson’s and their families in sub-Saharan Africa, through exploring care provision and receipt in Kenya. Chronic and progressive conditions, like Parkinson’s, challenge older people’s sense of self and identity. As care needs became more complex, families in this study contended with social change, taboos and avoidance rules, and tensions in reciprocity and resources in order to make care work. Crucially, what this research has demonstrated is the adaptability, resilience and enduring obligations of families to enact and reciprocate care, albeit in potentially less ‘conventional’ ways. Such practices involved care from a distance, relocations (decisions which could often be made for older PwP), and the formation of multigenerational urban households made up of unconventional co-residence.

Though this research on Parkinson’s is not representative of all older people in Kenya, it provides critical reflection on the care needs of older persons ageing with, and into, disability. Further work in Kenya, and elsewhere in Africa, is needed to build a stronger picture of how urban and rural families are contending with the challenges described through this paper, and how policy intends to support, sustain and strengthen community-based care for older people. This need is particularly acute given the growing and ageing population and accompanying ‘care deficit’.

## Data Availability

The qualitative data generated and analysed during the current study are not publicly available as participants did not consent to data sharing and doing so could compromise anonymity and confidentiality. Data are available from the corresponding author on reasonable request.

## References

[1] Dorsey ER, Elbaz A, Nichols E, Abd-Allah F, Abdelalim A, Adsuar JC, et al. Global, regional, and national burden of Parkinson’s disease, 1990–2016: a systematic analysis for the Global Burden of Disease Study 2016. Lancet Neurol. 2018;17(11):939–53. 10.1016/S1474-4422(18)30295-3 PMC6191528 30287051

[2] Dorsey ER, Sherer T, Okun MS, Bloem BR. The Emerging Evidence of the Parkinson Pandemic. J Parkinsons Dis. 2018;8(S1):S3–8. 10.3233/JPD-181474 PMC6311367 30584159

[3] DeMaagd G, Philip A. Parkinson’s Disease and Its Management: Part 1: Disease Entity, Risk Factors, Pathophysiology, Clinical Presentation, and Diagnosis. P T. 2015;40(8):504–32. PMC4517533 26236139

[4] World Health Organization. Global status report on neurology. Geneva; 2025.

[5] WHO. Atlas: country resources for neurological disorders – 2nd ed. Geneva: World Health Organization,; 2017.

[6] Fothergill-Misbah N, Cataldi R, Stothard M, Chowdhary N, Okubadejo N, Cappello B, et al. Parkinson disease treatments on national essential medicines lists, African Region. Bull World Health Organ. 2025;103(10):626–34. 10.2471/BLT.25.293460 PMC12481222 41035551

[7] Fothergill-Misbah N, Hooker J, Kwasa J, Walker R. Access to Medicines for Parkinson’s Disease in Kenya: A Qualitative Exploration. Mov Disorders Clin Pract. 2024;11(11):1373–8. 10.1002/mdc3.14192 PMC11542282 39166895

[8] Fothergill-Misbah N, Walker R, Kwasa J, Hooker J, Hampshire K. Old people problems, uncertainty and legitimacy: Challenges with diagnosing Parkinson’s disease in Kenya. Soc Sci Med. 2021;282:114148. 10.1016/j.socscimed.2021.114148 34153822

[9] Fothergill-Misbah N. The lived experience of stigma and parkinson’s disease in Kenya: a public health challenge. BMC Public Health. 2023;23(1):364. 10.1186/s12889-023-15278-7 PMC9940067 36803768

[10] HelpAge International. Older women’s lived experiences of gendered ageism. London: HelpAge International; 2023.

[11] HelpAge International. Older people in Kenya must be protected from witchcraft accusations: HelpAge International. 2021 [Available from: https://www.helpage.org/news/older-people-must-be-protected-from-witchcraft-accusations/. Access date 24/07/2026.

[12] United Nations Department of Economic and Social Affairs. World Population Prospects 2024, Download Files 2024 [Available from: https://population.un.org/wpp/Download/Standard/Population/. Access date 24/07/2026.

[13] Gyasi RM, Phillips DR. Aging and the Rising Burden of Noncommunicable Diseases in Sub-Saharan Africa and other Low- and Middle-Income Countries: A Call for Holistic Action. Gerontologist. 2019;60(5):806–11. 10.1093/geront/gnz102 31322656

[14] Apt N. Rapid Urbanization and Living Arrangements of Older Persons in Africa. UN Secretariat, Affairs EaS;; 2001.

[15] Awumbila M. Drivers of migration and urbanization in Africa: Key trends and issues. New York: UN Department of Economic and Social Affairs; 2017.

[16] Schatz E, Seeley J. Gender, ageing and carework in East and Southern Africa: A review. Glob Public Health. 2015;10(10):1185–200. 10.1080/17441692.2015.1035664 PMC4888771 25947225

[17] World Health O. Towards long-term care systems in sub-Saharan Africa. Geneva: World Health Organization; 2017 2017.

[18] KNBS. 2019 Kenya Population and Housing Census Volume III: Distribution of Population by Age and Sex. Nairobi; 2019a.

[19] Wan H, Aboderin I, Adjaye-Gbewonyo D, Bureau. UC. International Population Reports, P95/20 – 1 Africa Aging: 2020. Washington, DC.: U.S. Government Printing Office; 2020.

[20] World Health Organization. Measuring the progress and impact of the UK Decade of Healthy Ageing (2021–2030): framework and indicators recommended by WHO Technical Advisory Group. Geneva; 2024.

[21] Saka S, Oosthuizen F, Nlooto M. National Policies and Older People’s Healthcare in Sub-Saharan Africa: A Scoping Review. Ann Glob Health. 2019;85(1). 10.5334/aogh.2401 PMC6634477 31251482

[22] Republic of Kenya Ministry of Gender C, and, Services C. National Care Policy. 2025.

[23] McQuaid K, Esson J, Gough KV, Wignall R. Navigating old age and the urban terrain: Geographies of ageing from Africa. Prog Hum Geogr. 2021;45(4):814–33.

[24] Gouda H, Charlson F, Sorsdahl K, Ahmadzada S, Ferrari A, Erskine H, et al. Burden of non-communicable diseases in sub-Saharan Africa, 1990–2017: results from the Global Burden of Disease Study 2017. Lancet Global Health. 2019;7(10):e1375–87. 10.1016/S2214-109X(19)30374-2 31537368

[25] Steinmetz JD, Seeher KM, Schiess N, Nichols E, Cao B, Servili C, et al. Global, regional, and national burden of disorders affecting the nervous system, 1990–2021: a systematic analysis for the Global Burden of Disease Study 2021. Lancet Neurol. 2024;23(4):344–81. 10.1016/S1474-4422(24)00038-3 PMC10949203 38493795

[26] Li M, Ye X, Huang Z, Ye L, Chen C. Global burden of Parkinson’s disease from 1990 to 2021: a population-based study. BMJ Open. 2025;15(4):e095610. 10.1136/bmjopen-2024-095610 PMC12035419 40288800

[27] Su D, Cui Y, He C, Yin P, Bai R, Zhu J, et al. Projections for prevalence of Parkinson’s disease and its driving factors in 195 countries and territories to 2050: modelling study of Global Burden of Disease Study 2021. BMJ. 2025;388:e080952. 10.1136/bmj-2024-080952 PMC11881235 40044233

[28] Walker R, Fothergill-Misbah N, Kariuki S, Ojo O, Cilia R, Dekker MCJ, et al. Transforming Parkinson’s Care in Africa (TraPCAf): protocol for a multimethodology National Institute for Health and Care Research Global Health Research Group project. BMC Neurol. 2023;23(1):373. 10.1186/s12883-023-03414-0 PMC10585779 37858118

[29] Kuh D. A Life Course Approach to Healthy Ageing. In: Michel J-P, editor. Prevention of Chronic Diseases and Age-Related Disability. Cham: Springer International Publishing; 2019. pp. 1–9.

[30] World Health Organization. Life expectancy and Healthy life expectancy Data by WHO region: World Health Organization. 2020 [Available from: https://www.who.int/data/gho/data/themes/mortality-and-global-health-estimates/ghe-life-expectancy-and-healthy-life-expectancy. Access date 24/07/2026.

[31] Ferrari AJ, Santomauro DF, Aali A, Abate YH, Abbafati C, Abbastabar H, et al. Global incidence, prevalence, years lived with disability (YLDs), disability-adjusted life-years (DALYs), and healthy life expectancy (HALE) for 371 diseases and injuries in 204 countries and territories and 811 subnational locations, 1990–2013;2021: a systematic analysis for the Global Burden of Disease Study 2021. Lancet. 2024;403(10440):2133–61. 10.1016/S0140-6736(24)00757-8 PMC11122111 38642570

[32] International Labour Organization. Care work and care jobs for the future of decent work. Geneva; 2018.

[33] Hailu GN, Gebru HB, Hagos GG, Weldemariam AH, Tadesse DB, Mebrahtom G. The role of family caregivers in supporting older adults in Africa: systematic review. BMC Geriatr. 2025;25(1):491. 10.1186/s12877-025-06154-7 PMC12231671 40610950

[34] Mwendwa P, Lawlor B, Kroll T, De Brún A. A systematic review and narrative synthesis of the experiences with caring for older people living with dementia in Sub-Saharan Africa. BMC Geriatr. 2022;22(1):961. 10.1186/s12877-022-03668-2 PMC9749146 36514016

[35] Aboderin I, Hoffman J, Families. Intergenerational Bonds, and Aging in Sub-Saharan Africa. Can J Aging. 2015;34(3):282–9. 10.1017/S0714980815000239 26300188

[36] World Health Organization. Towards long-term care systems in sub-Saharan Africa. Geneva: World Health Organization; 2017.

[37] van Der Geest S. Will families in Ghana continue to care for older people? Logic and contradiction in policy. In: Hoffman J, Pype K, editors. Ageing in Sub-Saharan Africa: Spaces and Practices of Care. Policy. 2016;21–41. 10.2307/j.ctt1t89ckt.8.

[38] McIntosh J. Depreciating Age, Disintegrating Ties: On Being Old in a Century of Declining Elderhood in Kenya. In: Lamb S, editor. Successful Aging as a Contemporary Obsession: Global Perspectives. Rutgers University Press; 2017. pp. 185–99.

[39] Whyte SR. Successful Aging and Desired Interdependence. In: Lamb S, editor. Successful Aging as a Contemporary Obsession: Global Perspectives. Rutgers University Press; 2017. pp. 243–8.

[40] Kimuna SR. Living Arrangements and Conditions of Older People in Zimbabwe. Afr Popul Stud. 2005;20(2):146–63.

[41] Aboderin I. Decline in Material Family Support for Older People in Urban Ghana, Africa: Understanding Processes and Causes of Change. Journals Gerontology: Ser B. 2004;59(3):S128–37. 10.1093/geronb/59.3.s128 15118018

[42] Cattell M. Aging and Social Change Among Abaluyia in Western Kenya: Anthropological and Historical Perspectives. J Cross Cult Gerontol. 2008;23:181–97. 10.1007/s10823-008-9062-x 18386171

[43] Hoffman J, Pype K. Introduction: Spaces and practices of care for older people in Sub-Saharan Africa. In: Hoffman J, Pype K, editors. Ageing in Sub-Saharan Africa: Spaces and practices of care. Policy. 2016;1–20. 10.2307/j.ctt1t89ckt.7.

[44] Aboderin I. Modernisation and ageing theory revisited: current explanations of recent developing world and historical Western shifts in material family support for older people. Aging Soc. 2004;24(1):29–50.

[45] Nzima D, Maharaj P. Long-term care for the elderly in sub-Saharan Africa: an analysis of existing models. In: Maharaj P, editor. Health and Care in Old Age in Africa. London: Routledge; 2020.

[46] Obrist B. Place matters: the home as a key site of old-age care in coastal Tanzania. In: Hoffman J, Pype K, editors. Ageing in sub-Saharan Africa: Spaces and practices of care. Policy; 2016. pp. 95–114.

[47] van der Geest S. Respect and reciprocity: Care of elderly people in rural Ghana. J Cross-Cult Gerontol. 2002;17(1):3–31. 10.1023/a:1014843004627 14617973

[48] Rishworth A, Elliott S. Ageing in low- and muddle-income countries: ageing against all odds. In: Skinner M, Andrews G, Cutchin M, editors. Geographical Gerontology: Perspectives, Concepts, Approaches. Abingdon: Routledge; 2018.

[49] Reeves S, Kuper A, Hodges BD. Qualitative research methodologies: ethnography. BMJ. 2008;337(a1020):512–4. 10.1136/bmj.a1020 18687725

[50] Fetterman DM, Ethnography. Step-by-Step. 4th ed. SAGE; 2019.

[51] Braun V, Clarke V. Thematic Analysis: A Practical Guide. United Kingdom: SAGE; 2021.

[52] Arber A, Reflexivity. A Challenge for the Researcher as Practitioner? J Res Nurs. 2006;11(2):147–57.

[53] Charmaz K. 'Loss of self: a fundamental form of suffering in the chronically ill', Sociology of Health & Illness. 1983;5(2):168–95. 10.1111/1467-9566.ep10491512. 10261981

[54] Whyte SR. Chronicity and control: framing ‘noncommunicable diseases’ in Africa. Anthropol Med. 2012;19(1):63–74. 10.1080/13648470.2012.660465 22612492

[55] Walga TK. Understanding the Experience and Perspectives of Parkinson’s Disease Patients’ Caregivers. Rehabilitation Res Pract. 2019;2019:1–9. 10.1155/2019/3082325 PMC6374795 30838135

[56] McLaughlin D, Hasson F, Kernohan WG, Waldron M, McLaughlin M, Cochrane B, et al. Living and coping with Parkinson’s disease: perceptions of informal carers. Palliat Med. 2011;25(2):177–82. 10.1177/0269216310385604 20952448

[57] Tan SB, Williams AF, Morris ME. Experiences of caregivers of people with Parkinson’s disease in Singapore: a qualitative analysis. J Clin Nurs. 2012;21(15–16):2235–46. 10.1111/j.1365-2702.2012.04146.x 22788558

[58] Bhimani R. Understanding the Burden on Caregivers of People with Parkinson’s: A Scoping Review of the Literature. Rehabil Res Pract. 2014;2014:718527. 10.1155/2014/718527 PMC4179947 25298895

[59] Mosley PE, Moodie R, Dissanayaka N. Caregiver Burden in Parkinson Disease: A Critical Review of Recent Literature. J Geriatr Psychiatr Neurol. 2017;30(5):235–52. 10.1177/0891988717720302 28743212

[60] Apt N. Ageing and the changing role of the family and the community: An African Perspective. Int Social Secur Rev. 2002;55(1):39–47.

[61] Moore E, Samodien Z, Megannon V. Family Caregiving of Older Persons in South Africa Report. University of Cape Town; 2025.

[62] Golaz V, Wandera SO, Rutaremwa G. Understanding the vulnerability of older adults: extent of and breaches in support systems in Uganda. Aging Soc. 2017;37(1):63–89.

[63] van der Geest S. They don’t come to listen: the experience of loneliness among older people in Kwahu, Ghana. J Cross Cult Gerontol. 2004a;19(2):77–96. 10.1023/B:JCCG.0000027846.67305.f0 15146139

[64] Dintwat K. Changing Family Structure in Botswana. J Comp Family Stud. 2010;41:281–97.

[65] Hillcoat-Nalletamby S, Ogg JIM. Moving beyond ‘ageing in place’: older people’s dislikes about their home and neighbourhood environments as a motive for wishing to move. Aging Soc. 2014;34(10):1771–96.

[66] Cadmus EO, Adebusoye LA, Owoaje ET. Ageing in place or stuck in place: Preferred care setting for community-dwelling older persons in a low-resource country in Sub Saharan Africa. PLoS ONE. 2023;18(10):e0292939. 10.1371/journal.pone.0292939 PMC10578603 37844096

[67] Barrientos A, Gorman M, Heslop A. Old Age Poverty in Developing Countries: Contributions and Dependence in Later Life. World Dev. 2003;31(3):555–70.

[68] Niño-Zarazúa M, Barrientos A, Hickey S, Hulme D. Social Protection in Sub-Saharan Africa: Getting the Politics Right. World Dev. 2012;40(1):163–76.

[69] Lamb S. In/dependence, Intergenerational Uncertainty, and the Ambivalent State: Perceptions of Old Age Security in India. South Asia: J South Asian Stud. 2013;36(1):65–78.

[70] Akinyemi RO, Yaria J, Ojagbemi A, Guerchet M, Okubadejo N, Njamnshi AK, et al. Dementia in Africa: Current evidence, knowledge gaps, and future directions. Alzheimer’s Dement. 2022;18(4):790–809. 10.1002/alz.12432 PMC8957626 34569714

[71] Akinyemi RO, Ovbiagele B, Adeniji OA, Sarfo FS, Abd-Allah F, Adoukonou T, et al. Stroke in Africa: profile, progress, prospects and priorities. Nat Reviews Neurol. 2021;17(10):634–56. 10.1038/s41582-021-00542-4 PMC8441961 34526674

[72] Pilleron S, Soerjomataram I, Charvat H, Chokunonga E, Somdyala NIM, Wabinga H, et al. Cancer incidence in older adults in selected regions of sub-Saharan Africa, 2008–2012. Int J Cancer. 2019;144(8):1824–33. 10.1002/ijc.31880 30238972

[73] Kasa AS, Lee S-C, Chang H-C. Frailty in older people living in Africa: A systematic review of prevalence and associated factors. Archives Gerontol Geriatr Plus. 2024;1(4):100078.

[74] Ministry of Labour SSaS. National Policy on Older Persons and Ageing. 2018.

